# Characteristics of gambling among Tunisian students

**DOI:** 10.1192/j.eurpsy.2025.952

**Published:** 2025-08-26

**Authors:** R. Jbir, R. Masmoudi, F. Cherif, M. Abdelkefi, I. Feki, I. Baati, J. Masmoudi

**Affiliations:** 1 psychiatry ‘A’ department, Hedi Chaker university hospital, sfax, Tunisia

## Abstract

**Introduction:**

Nowadays, gambling has become a common form of entertainment for many young people. Gambling is essentially divided into dice games, card games (especially poker), casino games, sports betting, lotteries and, in recent years, online gambling. As a result, gambling has become a major international business, especially among young students.

**Objectives:**

Evaluate the practice of gambling in a population of young students and describe its characteristics.

**Methods:**

We conducted a descriptive and analytical cross-sectional study among university students.

This survey involved a population of Tunisian students recruited through students’ Facebook groups.

A questionnaire was designed to collect information related to participants’ background characteristics and gambling-related details.

DSM-5 criteria were used to screen for pathological gamblers.

**Results:**

A total of 151 students responded to our questionnaire. Gambling was found in 29.1% of the students in our study (n=44).

Thirty players (68.4%) were occasional players. The lottery (54.5%), followed by sports betting (29.5%) and blackjack (27.3%) were the most reported gambling games by students.

Among the players, nineteen (43.2%) experienced a Big Win. The average amount of money spent on gambling per month was 107.14 ± 123.58 TND, with extremes of 4 and 600 TND.

The majority (77.3%; N=34) played online. Among players, 61.4% (N=27) were used to play with friends.

Almost the half of gamblers (45.5%) have started gambling for less than a year and 6.8% have been gambling for 4 to 5 years.

Pathological gamblers represented 13.9% (n=21) of the students in our population.

**Conclusions:**

Our study highlights the prevalence and characteristics of gambling among university students in Tunisia. With nearly 30% of participants engaging in various forms of gambling. These findings suggest a need for targeted awareness and prevention strategies to address potential risks associated with gambling among young adults, particularly in educational settings. Further researches are essential to explore the long-term implications of gambling behaviors on student well-being and academic performance.

**Disclosure of Interest:**

None Declared

